# [3-Benzoyl-2,4-bis­(3-nitro­phen­yl)cyclo­but­yl](phen­yl)methanone

**DOI:** 10.1107/S1600536812044650

**Published:** 2012-11-03

**Authors:** Prakash S. Nayak, Badiadka Narayana, Hemmige S. Yathirajan, Thomas Gerber, Eric Hosten, Richard Betz

**Affiliations:** a Mangalore University, Department of Studies in Chemistry, Mangalagangotri 574 199, India; b University of Mysore, Department of Studies in Chemistry, Manasagangotri, Mysore 570 006, India; c Nelson Mandela Metropolitan University, Summerstrand Campus, Department of Chemistry, University Way, Summerstrand, PO Box 77000, Port Elizabeth, 6031, South Africa

## Abstract

The asymmetric unit of the title compound, C_30_H_22_N_2_O_6_, comprises a half-mol­ecule of the cyclo­butane derivative. The least-squares planes defined by the respective C atoms of the aromatic substituents inter­sect at angles of 76.81 (7) and 89.22 (8)° with the least-squares plane defined by the C atoms of the cyclo­butane ring. In the crystal, C—H⋯O contacts connect the mol­ecules into a three-dimensional network. The shortest centroid–centroid distance between the two different aromatic rings is 3.9601 (8) Å.

## Related literature

For the biological activity of chalcones and cyclo­butane-derived compounds, see: Dimmock *et al.* (1999 ▶); Marais *et al.* (2005 ▶); Katerere *et al.* (2004 ▶); Seidel *et al.* (2000 ▶). For the crystal structures of similar compounds, see: Zheng *et al.* (2001 ▶); Zhuang & Zheng (2002 ▶). For general information about the dimerization of chalcones, see: Stobbe & Bremer (1929 ▶); Mustafa (1952 ▶). For puckering analysis of cyclic motifs, see: Cremer & Pople (1975 ▶). For graph-set analysis of hydrogen bonds, see: Etter *et al.* (1990 ▶); Bernstein *et al.* (1995 ▶).
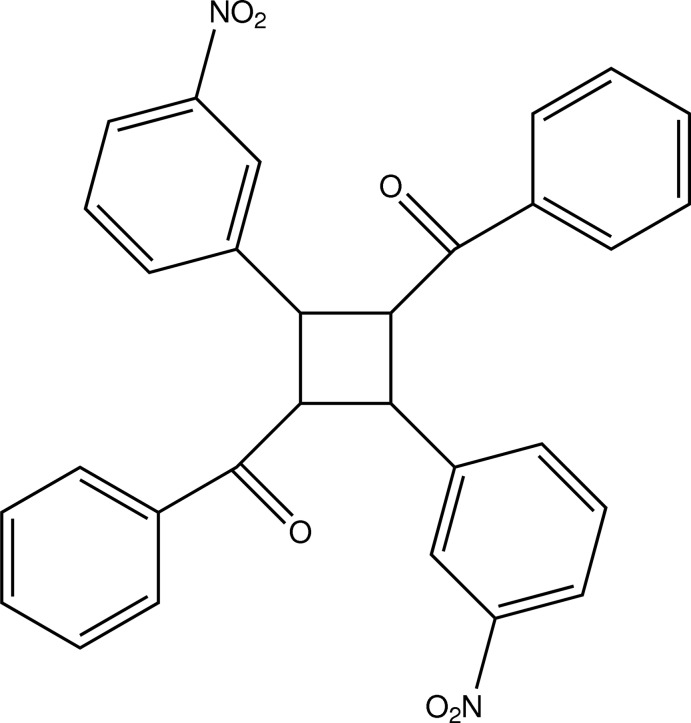


## Experimental

### 

#### Crystal data

C_30_H_22_N_2_O_6_
*M* *_r_* = 506.50
Monoclinic, 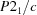

*a* = 5.7850 (1) Å
*b* = 14.7824 (3) Å
*c* = 14.3589 (3) Å
β = 104.858 (1)°
*V* = 1186.86 (4) Å^3^
*Z* = 2
Mo *K*α radiation
μ = 0.10 mm^−1^
*T* = 200 K
0.33 × 0.14 × 0.11 mm

#### Data collection

Bruker APEXII CCD diffractometer
Absorption correction: multi-scan (*SADABS*; Bruker, 2008 ▶) *T*_min_ = 0.968, *T*_max_ = 0.989
11005 measured reflections
2945 independent reflections
2387 reflections with *I* > 2σ(*I*)
*R*_int_ = 0.020

#### Refinement

*R*[*F* ^2^ > 2σ(*F* ^2^)] = 0.040
*wR*(*F* ^2^) = 0.111
*S* = 1.03
2945 reflections
172 parameters
H-atom parameters constrained
Δρ_max_ = 0.32 e Å^−3^
Δρ_min_ = −0.22 e Å^−3^

### 

Data collection: *APEX2* (Bruker, 2010 ▶); cell refinement: *SAINT* (Bruker, 2010 ▶); data reduction: *SAINT*; program(s) used to solve structure: *SHELXS97* (Sheldrick, 2008 ▶); program(s) used to refine structure: *SHELXL97* (Sheldrick, 2008 ▶); molecular graphics: *ORTEP-3* (Farrugia, 2012 ▶) and *Mercury* (Macrae *et al.*, 2008 ▶); software used to prepare material for publication: *SHELXL97* and *PLATON* (Spek, 2009 ▶).

## Supplementary Material

Crystal structure: contains datablock(s) I, global. DOI: 10.1107/S1600536812044650/fj2603sup1.cif

Supplementary material file. DOI: 10.1107/S1600536812044650/fj2603Isup2.cdx

Structure factors: contains datablock(s) I. DOI: 10.1107/S1600536812044650/fj2603Isup3.hkl

Supplementary material file. DOI: 10.1107/S1600536812044650/fj2603Isup4.cml

Additional supplementary materials:  crystallographic information; 3D view; checkCIF report

## Figures and Tables

**Table 1:** Hydrogen-bond geometry (Å, °)

*D*—H⋯*A*	*D*—H	H⋯*A*	*D*⋯*A*	*D*—H⋯*A*
C3—H3⋯O1^i^	1.00	2.56	3.3957 (15)	141
C14—H14⋯O2^ii^	0.95	2.56	3.3666 (19)	142
C2—H2⋯O3^iii^	1.00	2.61	3.5009 (17)	148

Symmetry codes: (i) 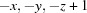; (ii) 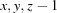; (iii) 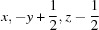.

**Fig. 1.**
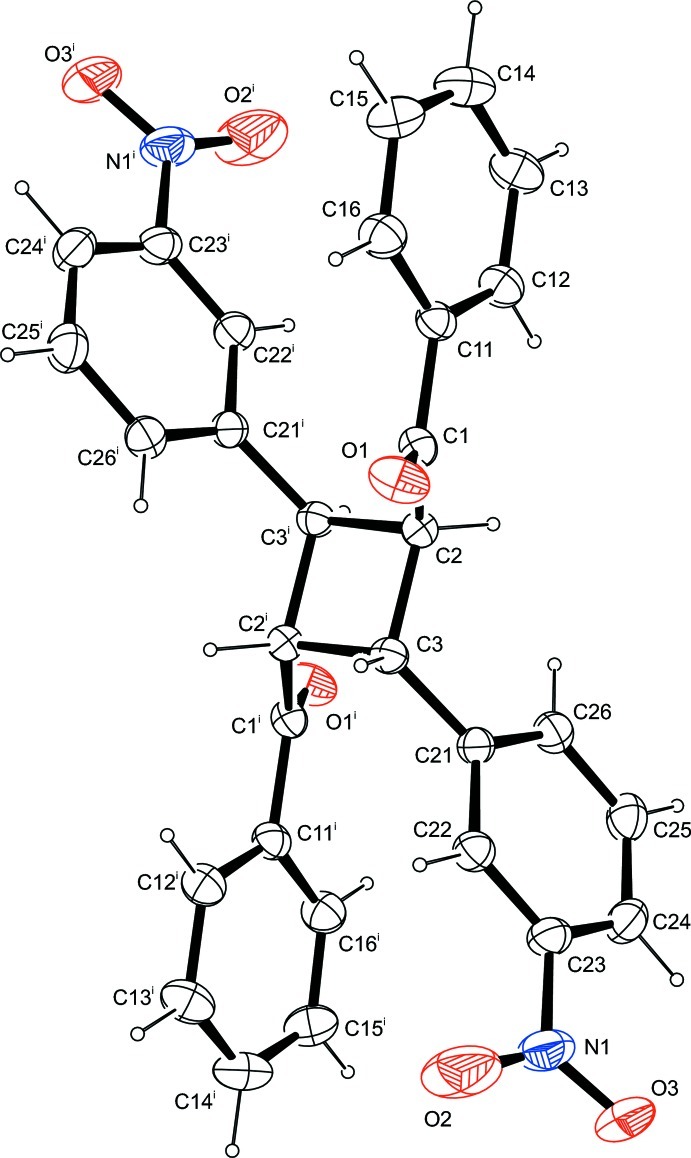
The molecular structure of the title compound, with atom labels and anisotropic displacement ellipsoids (drawn at 50% probability level). Symmetry operator: i -x + 1, -y, -z + 1.

**Fig. 2.**
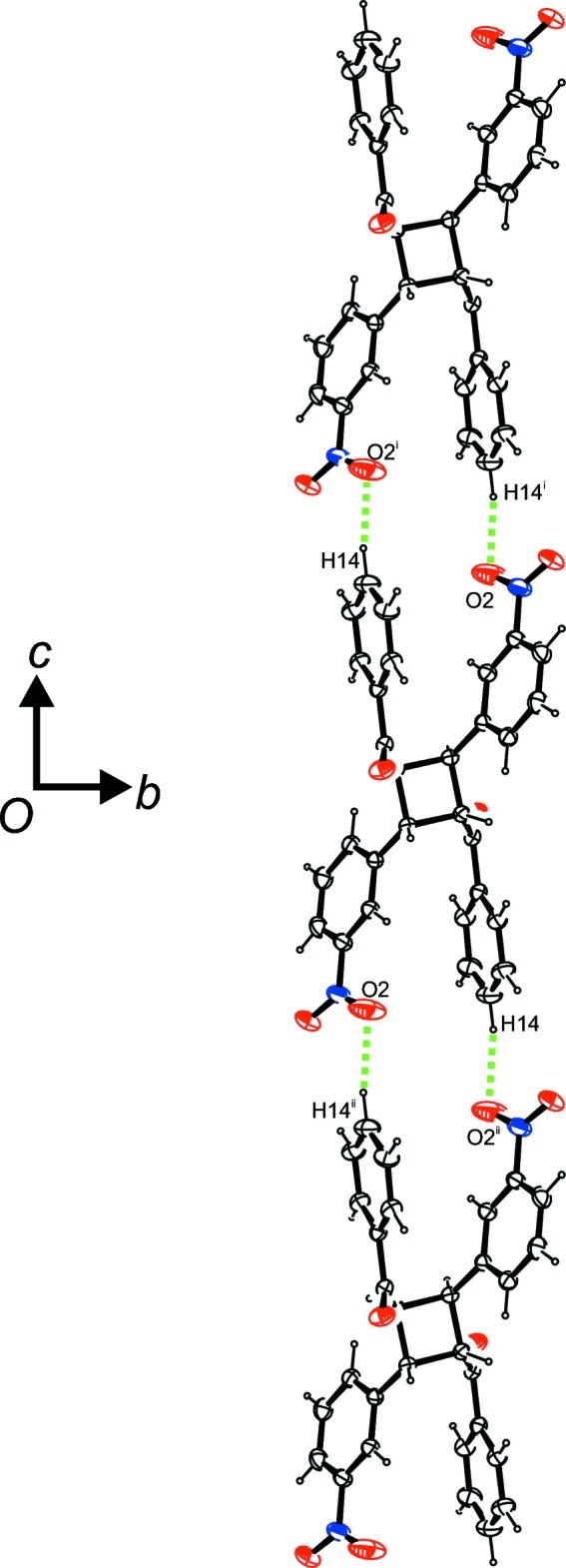
Intermolecular contacts, viewed along [-1 0 0]. For clarity, only a selection of contacts is depicted. Symmetry operators: ix, y, z - 1; iix, y, z + 1.

**Fig. 3.**
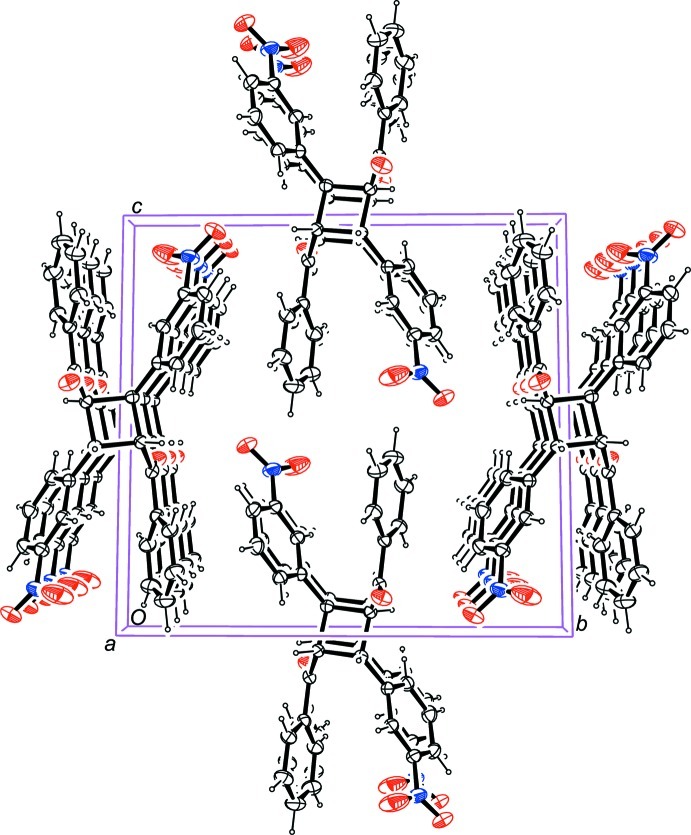
Molecular packing of the title compound, viewed along [-1 0 0] (anisotropic displacement ellipsoids drawn at 50% probability level).

## supplementary crystallographic information

### Comment

Chalcones comprise one of the most commonly occurring classes of medicinally important natural compounds, since they show various biological activities (Dimmock *et al.*, 1999; Marais *et al.*,. 2005). Cyclobutane-containing natural products have, *e.g.*, been reported for Combretum albopunctatum (Katerere *et al.*, 2004) and Goniothalamus thwaitesii (Seidel *et al.* 2000). Because of the various biological activities of these natural compounds, the synthesis of cyclobutane-derived compounds is one of the most intensively studied photochemical reactions of chalcone derivatives. These reactions can be carried out in solution, solid state and molten state by sunlight or UV-vis irradiation, with variable results in terms of yield and product composition (Stobbe & Bremer, 1929; Mustafa, 1952). The crystal structures of some dimerized chalcones such as *r*-1,*c*-2,*t*-3,*t*-4- 1,3-bis(4-methoxyphenyl)-2,4-bis(5-phenyl-1,3,4-oxadiazol-2-yl) cyclobutane 1,4-dioxane solvate (Zheng *et al.*, 2001) and *r*-1,*c*-2,*t*- 3,*t*-4–1,2-bis(4-methoxyphenyl)-3,4-bis(5-phenyl-1,3,4-oxadiazol-2-yl) cyclobutane (Zhuang & Zheng, 2002) have been reported. In view of the pharmacological importance of chalcone derivatives, the synthesis of such a compound was attempted. Upon the determination of the reaction product's crystal structure, the unintentional formation of the corresponding dimer in the wake of the reaction sequence was revealed.

The title compound, [3-benzoyl-2,4-bis(3-nitrophenyl)cyclobutyl](phenyl)methanone, features a central cyclobutane moiety that bears one aromatic substituent on each carbon atom. Due to the centrosymmetry of the molecule, the relative orientation of these substituents corresponds to *cis*-*trans*-*cis*-*trans*. The small puckering amplitude precludes a puckering analysis of this ring (Cremer & Pople, 1975). The least-squares planes defined by the respective carbon atoms of the aromatic substituents intersect with the least-squares plane defined the carbon atoms of the cyclobutane ring at angles of 76.81 (7) ° and 89.22 (8) °. The aforementioned planes of the two different aromatic moieties in the asymmetric unit enclose an angle of 24.09 (6) ° (Fig. 1).

In the crystal, intermolecular C–H···O contacts whose range falls by more than 0.1 Å below the sum of van-der-Waals radii of the respective atoms can be observed. These are supported by the hydrogen atom in *para* position of the non-substituted phenyl group as well as all methine-type hydrogen atoms while the hydrogen atoms of the nitrophenyl moiety do not take part in such contacts. All oxygen atoms present in the molecule act as acceptors. Furthermore, one intramolecular C–H···O contact between a carbonyl group and a methine-type hydrogen atom is apparent. In total, the molecules are connected to a three-dimensional network. In terms of graph-set analysis (Etter *et al.*, 1990; Bernstein *et al.*, 1995), the descriptor for these contacts is *S*(5)*C*^1^_1_(8)*C*^1^_1_(13)*R*^2^_2_(10) on the unary level. Metrical parameters as well as information about the symmetry of these contacts are summarized in Table 1. The shortest intercentroid distance between two aromatic systems was measured at 3.9601 (8) Å and is apparent between the two different aromatic substituents (Fig. 2).

The packing of the title compound in the crystal structure is shown in Figure 3.

### Experimental

To a mixture of 3-nitrobenzaldehyde (1.51 g, 0.01 mol) and acetophenone (1.16 ml, 0.01 mol) in ethanol (50 ml), a sodium hydroxide solution (10%, 10 ml) was added. The mixture was stirred at 278–283 K for 3 h. The precipitate formed was collected by filtration and purified by recrystallization from ethanol. Single crystals suitable for the X-ray diffraction study were grown from methanol by slow evaporation at room temperature. The synthesized chalcone was dimerized during crystallization.

### Refinement

Carbon-bound H atoms were placed in calculated positions (C—H 0.95 Å for aromatic carbon atoms and C—H 1.00 Å for methine groups) and were included in the refinement in the riding model approximation, with *U*(H) set to 1.2*U*_eq_(C).

### Crystal data

**Table:**

C_30_H_22_N_2_O_6_	*F*(000) = 528
*M**_r_* = 506.50	*D*_x_ = 1.417 Mg m^−^^3^
Monoclinic, *P*2_1_/*c*	Melting point > 523 K
Hall symbol: -P 2ybc	Mo *K*α radiation, λ = 0.71073 Å
*a* = 5.7850 (1) Å	Cell parameters from 4849 reflections
*b* = 14.7824 (3) Å	θ = 2.8–28.3°
*c* = 14.3589 (3) Å	µ = 0.10 mm^−^^1^
β = 104.858 (1)°	*T* = 200 K
*V* = 1186.86 (4) Å^3^	Block, colourless
*Z* = 2	0.33 × 0.14 × 0.11 mm

### Data collection

**Table:**

Bruker APEXII CCD diffractometer	2945 independent reflections
Radiation source: fine-focus sealed tube	2387 reflections with *I* > 2σ(*I*)
Graphite monochromator	*R*_int_ = 0.020
φ and ω scans	θ_max_ = 28.5°, θ_min_ = 2.8°
Absorption correction: multi-scan (*SADABS*; Bruker, 2008)	*h* = −7→7
*T*_min_ = 0.968, *T*_max_ = 0.989	*k* = −17→19
11005 measured reflections	*l* = −17→19

### Refinement

**Table:**

Refinement on *F*^2^	Primary atom site location: structure-invariant direct methods
Least-squares matrix: full	Secondary atom site location: difference Fourier map
*R*[*F*^2^ > 2σ(*F*^2^)] = 0.040	Hydrogen site location: inferred from neighbouring sites
*wR*(*F*^2^) = 0.111	H-atom parameters constrained
*S* = 1.03	*w* = 1/[σ^2^(*F*_o_^2^) + (0.055*P*)^2^ + 0.3935*P*] where *P* = (*F*_o_^2^ + 2*F*_c_^2^)/3
2945 reflections	(Δ/σ)_max_ < 0.001
172 parameters	Δρ_max_ = 0.32 e Å^−^^3^
0 restraints	Δρ_min_ = −0.22 e Å^−^^3^

### Fractional atomic coordinates and isotropic or equivalent isotropic displacement parameters (Å^2^)

**Table:**

	*x*	*y*	*z*	*U*_iso_*/*U*_eq_	
O1	0.02716 (16)	0.08341 (7)	0.41101 (7)	0.0343 (2)	
O2	0.4112 (3)	0.11125 (12)	0.89447 (9)	0.0693 (4)	
O3	0.6594 (3)	0.21980 (8)	0.94461 (8)	0.0584 (4)	
N1	0.5674 (2)	0.16321 (9)	0.88471 (8)	0.0386 (3)	
C1	0.2098 (2)	0.07918 (8)	0.38436 (8)	0.0223 (2)	
C2	0.4494 (2)	0.05901 (8)	0.45303 (8)	0.0206 (2)	
H2	0.5684	0.1074	0.4506	0.025*	
C3	0.44539 (19)	0.03813 (8)	0.55776 (8)	0.0203 (2)	
H3	0.2767	0.0331	0.5629	0.024*	
C11	0.2032 (2)	0.09061 (8)	0.28021 (8)	0.0224 (2)	
C12	0.3971 (2)	0.12395 (9)	0.24994 (9)	0.0266 (3)	
H12	0.5414	0.1391	0.2960	0.032*	
C13	0.3792 (2)	0.13495 (10)	0.15237 (9)	0.0333 (3)	
H13	0.5096	0.1596	0.1318	0.040*	
C14	0.1718 (3)	0.11009 (11)	0.08487 (10)	0.0371 (3)	
H14	0.1607	0.1173	0.0181	0.045*	
C15	−0.0192 (2)	0.07487 (10)	0.11440 (9)	0.0358 (3)	
H15	−0.1599	0.0565	0.0680	0.043*	
C16	−0.0049 (2)	0.06645 (9)	0.21178 (9)	0.0287 (3)	
H16	−0.1381	0.0440	0.2320	0.034*	
C21	0.5883 (2)	0.09762 (8)	0.63665 (8)	0.0209 (2)	
C22	0.5151 (2)	0.10549 (8)	0.72138 (8)	0.0245 (2)	
H22	0.3736	0.0762	0.7275	0.029*	
C23	0.6502 (2)	0.15632 (8)	0.79654 (9)	0.0276 (3)	
C24	0.8559 (2)	0.20118 (9)	0.79156 (9)	0.0319 (3)	
H24	0.9447	0.2362	0.8441	0.038*	
C25	0.9279 (2)	0.19330 (9)	0.70728 (10)	0.0307 (3)	
H25	1.0684	0.2235	0.7015	0.037*	
C26	0.7973 (2)	0.14165 (8)	0.63095 (9)	0.0254 (2)	
H26	0.8512	0.1363	0.5740	0.030*	

### Atomic displacement parameters (Å^2^)

**Table:**

	*U*^11^	*U*^22^	*U*^33^	*U*^12^	*U*^13^	*U*^23^
O1	0.0268 (4)	0.0478 (6)	0.0298 (5)	0.0065 (4)	0.0101 (4)	0.0091 (4)
O2	0.0738 (9)	0.1032 (12)	0.0425 (7)	−0.0292 (8)	0.0360 (6)	−0.0212 (7)
O3	0.1001 (10)	0.0466 (7)	0.0285 (5)	−0.0007 (7)	0.0169 (6)	−0.0144 (5)
N1	0.0505 (7)	0.0419 (7)	0.0239 (5)	0.0073 (6)	0.0106 (5)	−0.0050 (5)
C1	0.0250 (5)	0.0196 (5)	0.0222 (5)	0.0009 (4)	0.0059 (4)	0.0023 (4)
C2	0.0227 (5)	0.0199 (5)	0.0194 (5)	−0.0018 (4)	0.0059 (4)	0.0002 (4)
C3	0.0206 (5)	0.0219 (5)	0.0185 (5)	−0.0005 (4)	0.0056 (4)	0.0006 (4)
C11	0.0246 (5)	0.0210 (5)	0.0214 (5)	0.0035 (4)	0.0055 (4)	0.0027 (4)
C12	0.0244 (6)	0.0290 (6)	0.0265 (6)	0.0013 (5)	0.0064 (4)	0.0031 (5)
C13	0.0318 (6)	0.0419 (7)	0.0296 (6)	0.0041 (6)	0.0144 (5)	0.0068 (6)
C14	0.0405 (7)	0.0497 (8)	0.0218 (6)	0.0089 (6)	0.0091 (5)	0.0023 (6)
C15	0.0335 (7)	0.0455 (8)	0.0248 (6)	0.0003 (6)	0.0007 (5)	−0.0029 (6)
C16	0.0258 (6)	0.0320 (6)	0.0276 (6)	−0.0006 (5)	0.0052 (5)	0.0021 (5)
C21	0.0237 (5)	0.0190 (5)	0.0197 (5)	0.0025 (4)	0.0050 (4)	0.0000 (4)
C22	0.0267 (6)	0.0241 (6)	0.0237 (5)	0.0019 (4)	0.0085 (4)	0.0000 (4)
C23	0.0355 (6)	0.0259 (6)	0.0213 (6)	0.0055 (5)	0.0073 (5)	−0.0017 (5)
C24	0.0373 (7)	0.0256 (6)	0.0280 (6)	−0.0008 (5)	−0.0004 (5)	−0.0050 (5)
C25	0.0284 (6)	0.0266 (6)	0.0354 (7)	−0.0052 (5)	0.0050 (5)	−0.0005 (5)
C26	0.0263 (6)	0.0244 (6)	0.0260 (6)	−0.0003 (4)	0.0076 (4)	0.0009 (5)

### Geometric parameters (Å, º)

**Table:**

O1—C1	1.2141 (15)	C13—H13	0.9500
O2—N1	1.2210 (19)	C14—C15	1.383 (2)
O3—N1	1.2199 (16)	C14—H14	0.9500
N1—C23	1.4676 (17)	C15—C16	1.3850 (18)
C1—C11	1.4957 (16)	C15—H15	0.9500
C1—C2	1.5104 (15)	C16—H16	0.9500
C2—C3	1.5412 (15)	C21—C22	1.3917 (16)
C2—C3^i^	1.5827 (16)	C21—C26	1.3938 (17)
C2—H2	1.0000	C22—C23	1.3810 (17)
C3—C21	1.5032 (15)	C22—H22	0.9500
C3—C2^i^	1.5827 (16)	C23—C24	1.380 (2)
C3—H3	1.0000	C24—C25	1.382 (2)
C11—C16	1.3910 (16)	C24—H24	0.9500
C11—C12	1.3931 (17)	C25—C26	1.3895 (17)
C12—C13	1.3877 (17)	C25—H25	0.9500
C12—H12	0.9500	C26—H26	0.9500
C13—C14	1.385 (2)		
			
O3—N1—O2	123.53 (13)	C15—C14—C13	120.16 (12)
O3—N1—C23	118.42 (13)	C15—C14—H14	119.9
O2—N1—C23	118.05 (12)	C13—C14—H14	119.9
O1—C1—C11	120.53 (10)	C14—C15—C16	119.85 (12)
O1—C1—C2	122.11 (10)	C14—C15—H15	120.1
C11—C1—C2	117.32 (10)	C16—C15—H15	120.1
C1—C2—C3	115.82 (9)	C15—C16—C11	120.46 (12)
C1—C2—C3^i^	115.26 (9)	C15—C16—H16	119.8
C3—C2—C3^i^	90.93 (8)	C11—C16—H16	119.8
C1—C2—H2	111.1	C22—C21—C26	118.37 (10)
C3—C2—H2	111.1	C22—C21—C3	118.42 (10)
C3^i^—C2—H2	111.1	C26—C21—C3	123.13 (10)
C21—C3—C2	118.37 (9)	C23—C22—C21	119.39 (12)
C21—C3—C2^i^	116.97 (9)	C23—C22—H22	120.3
C2—C3—C2^i^	89.07 (8)	C21—C22—H22	120.3
C21—C3—H3	110.3	C24—C23—C22	122.92 (12)
C2—C3—H3	110.3	C24—C23—N1	119.20 (11)
C2^i^—C3—H3	110.3	C22—C23—N1	117.87 (12)
C16—C11—C12	119.38 (11)	C23—C24—C25	117.54 (11)
C16—C11—C1	118.23 (11)	C23—C24—H24	121.2
C12—C11—C1	122.39 (10)	C25—C24—H24	121.2
C13—C12—C11	119.94 (11)	C24—C25—C26	120.79 (12)
C13—C12—H12	120.0	C24—C25—H25	119.6
C11—C12—H12	120.0	C26—C25—H25	119.6
C14—C13—C12	120.16 (12)	C25—C26—C21	120.99 (11)
C14—C13—H13	119.9	C25—C26—H26	119.5
C12—C13—H13	119.9	C21—C26—H26	119.5
			
O1—C1—C2—C3	3.75 (16)	C1—C11—C16—C15	−179.34 (12)
C11—C1—C2—C3	−174.02 (9)	C2—C3—C21—C22	153.77 (10)
O1—C1—C2—C3^i^	108.12 (13)	C2^i^—C3—C21—C22	−101.51 (12)
C11—C1—C2—C3^i^	−69.65 (13)	C2—C3—C21—C26	−29.57 (16)
C1—C2—C3—C21	−120.73 (11)	C2^i^—C3—C21—C26	75.16 (14)
C3^i^—C2—C3—C21	120.45 (11)	C26—C21—C22—C23	0.10 (17)
C1—C2—C3—C2^i^	118.82 (11)	C3—C21—C22—C23	176.93 (11)
C3^i^—C2—C3—C2^i^	0.0	C21—C22—C23—C24	0.70 (19)
O1—C1—C11—C16	−28.05 (17)	C21—C22—C23—N1	179.91 (11)
C2—C1—C11—C16	149.76 (11)	O3—N1—C23—C24	11.86 (19)
O1—C1—C11—C12	152.23 (12)	O2—N1—C23—C24	−167.48 (14)
C2—C1—C11—C12	−29.96 (16)	O3—N1—C23—C22	−167.39 (13)
C16—C11—C12—C13	1.65 (18)	O2—N1—C23—C22	13.3 (2)
C1—C11—C12—C13	−178.63 (12)	C22—C23—C24—C25	−0.62 (19)
C11—C12—C13—C14	−2.1 (2)	N1—C23—C24—C25	−179.82 (12)
C12—C13—C14—C15	0.5 (2)	C23—C24—C25—C26	−0.24 (19)
C13—C14—C15—C16	1.5 (2)	C24—C25—C26—C21	1.03 (19)
C14—C15—C16—C11	−2.0 (2)	C22—C21—C26—C25	−0.94 (18)
C12—C11—C16—C15	0.39 (19)	C3—C21—C26—C25	−177.61 (11)

Symmetry code: (i) −*x*+1, −*y*, −*z*+1.

### Hydrogen-bond geometry (Å, º)

**Table:**

*D*—H···*A*	*D*—H	H···*A*	*D*···*A*	*D*—H···*A*
C3—H3···O1	1.00	2.40	2.8509 (14)	106
C3—H3···O1^ii^	1.00	2.56	3.3957 (15)	141
C14—H14···O2^iii^	0.95	2.56	3.3666 (19)	142
C2—H2···O3^iv^	1.00	2.61	3.5009 (17)	148

Symmetry codes: (ii) −*x*, −*y*, −*z*+1; (iii) *x*, *y*, *z*−1; (iv) *x*, −*y*+1/2, *z*−1/2.
